# High-throughput phenotypic screening identifies the phytochemical melissic acid that mitigates SHED cellular senescence via the ADCY5/cAMP/CREB axis

**DOI:** 10.3389/fphar.2026.1908181

**Published:** 2026-09-17

**Authors:** Kanglu Yang, Lanyibo Liu, Caifeng Li, Shiwen Deng, Junxian Cao, Junqi Chen, Shaoping Wang, Qiangpu Chen, Peng Chen

**Affiliations:** 1 Shandong Medical and Pharmaceutical University Hospital, Shandong Medical and Pharmaceutical University, Binzhou, China; 2 Experimental Research Center, China Academy of Chinese Medical Sciences, Beijing, China; 3 Jiangxi Province Key Laboratory of Traditional Chinese Medicine Pharmacology, Institute of Traditional Chinese Medicine Health Industry, China Academy of Chinese Medical Sciences, Nanchang, China; 4 Analysis of Complex Effects of Proprietary Chinese Medicine, Hunan Provincial Key Laboratory, Yongzhou, Hunan Province, China

**Keywords:** ADCY5, autophagy, cellular senescence, melissic acid, phytochemical, SHED

## Abstract

**Introduction:**

Cellular senescence drives aging and age-related diseases, posing a critical barrier to stem cell-based regeneration. While selective elimination of senescent cells (senolysis) remains a theoretically promising strategy, safe natural senotherapeutic agents are limited. Human exfoliated deciduous tooth stem cells (SHED) possess high regenerative potential yet are vulnerable to senescence, compromising their therapeutic utility.

**Methods:**

We performed a phenotypic screen of 800 natural compounds in replicatively senescent SHED. The hit compound, the phytochemical melissic acid, was then validated in two independent senescence models—replicative exhaustion and doxorubicin-induced stress. Melissic acid (5–20 μM) was assessed for cytotoxicity and anti-senescence efficacy via SA-β-Gal staining and senescence marker expression. Transcriptomic profiling and mechanistic assays were performed to dissect the underlying molecular pathways.

**Results:**

Melissic acid exhibited minimal cytotoxicity but significantly reduced SA-β-Gal positivity and senescence markers in both models. Transcriptomic profiling revealed marked upregulation of ADCY5 and coordinated changes in cAMP signaling, autophagy, and lipid metabolism genes. Mechanistically, melissic acid increased CREB Ser133 phosphorylation, enhanced Beclin-1 expression, and reduced p62/SQSTM1, indicating activation of cAMP/CREB-dependent autophagy. It also decreased lipid peroxidation, restored mitochondrial membrane potential, and relieved G0/G1 arrest via upregulation of Cyclin D1 and Rb Ser780 phosphorylation.

**Discussion:**

Collectively, melissic acid alleviates SHED senescence through the ADCY5/cAMP/CREB-autophagy axis, promoting autophagic clearance, mitochondrial recovery, and cell-cycle re-entry—thereby ameliorating senescence-associated phenotypes in senescent stem cells. This study identifies melissic acid as a natural senomorphic candidate for stem cell function restoration and provides a mechanistic link between cAMP-dependent autophagy and amelioration of stem cell aging, offering a potential strategy to overcome stem cell exhaustion and regenerative limits imposed by cellular senescence.

## 1 Introduction

Cellular senescence is a dynamic cellular process characterized by a stable and essentially irreversible cell-cycle arrest, induced by diverse stresses, including telomere attrition, persistent DNA damage, oxidative stress, mitochondrial dysfunction, and aberrant oncogenic signaling. Senescent cells acquire a distinct phenotype characterized by enlarged and flattened morphology, increased SA-β-Gal activity, persistent DNA damage response activation, and a complex pro-inflammatory secretome known as the senescence-associated secretory phenotype (SASP) (Gorgoulis et al., 2019; Kirkland and Tchkonia, 2017). Although senescence serves as a potent tumor-suppressive mechanism under acute stress, the chronic accumulation of senescent cells in aging tissues disrupts tissue homeostasis, fuels chronic inflammation, and accelerates the progression of age-related degenerative disorders (López-Otín et al., 2023; Campisi, 2013). Consequently, the selective elimination of senescent cells has emerged as a promising strategy to promote healthy aging and mitigate age-related pathology (Niedernhofer and Robbins, 2018).

Accumulating evidence indicates that senescent cells are not merely dysfunctional bystanders but active drivers of tissue decline. In multiple preclinical models across different organ systems, clearance of senescent cells ameliorates age-associated phenotypes and improves organ function, positioning senotherapy as a promising translational anti-aging strategy (Justice et al., 2019; Xu et al., 2018). Nevertheless, the development of clinically applicable senotherapeutic agents remains challenging (Martel et al., 2020). Most currently described senolytic agents act by disrupting anti-apoptotic survival pathways, particularly the senescence-associated anti-apoptotic pathways (SCAPs) in senescent cells, thereby triggering apoptosis. Despite encouraging results, many of these compounds suffer from incomplete selectivity, suboptimal pharmacokinetics, and off-target effects *in vivo*. Hence, identifying anti-senescence compounds with distinct mechanisms of action and improved safety profiles for clinical translation remains an urgent priority (Zhu et al., 2017).

Phytochemicals and other natural products represent an attractive source of senotherapeutic compounds due to their structural diversity, broad pharmacological activities, favorable biocompatibility, and long history of human exposure. Several natural molecules, such as fisetin and quercetin, have been reported to exhibit anti-senescence activity; however, their mechanisms are often pleiotropic and incompletely defined (Yousefzadeh et al., 2018). Moreover, most known senolytic agents primarily induce cell death through apoptosis-related pathways via SCAP disruption, whereas relatively little is known about natural compounds that eliminate senescent cells by rewiring metabolic and organelle quality-control programs―a distinguishing feature of senomorphic agents (Kirkland et al., 2017). In this context, transcriptome-guided phenotypic screening provides a powerful strategy for identifying bioactive molecules and uncovering the signaling networks that underlie their activity (Wang et al., 2009).

Melissic acid (triacontanoic acid) is a phytochemical long-chain saturated fatty acid naturally present in beeswax, propolis, and plant cuticles. Previous studies have suggested that long-chain fatty acids participate in the regulation of inflammatory responses, oxidative homeostasis, and metabolic adaptation (Cornara et al., 2017; O’Neill et al., 2016). Because altered lipid metabolism, oxidative damage, and mitochondrial dysfunction are closely linked to cellular senescence, melissic acid represents a plausible candidate for senescence-targeted intervention. In this study, we performed a phenotypic screen of 800 natural compounds in replicatively senescent human exfoliated deciduous tooth stem cells (SHED) and identified melissic acid as a candidate anti-senescence compound. Its anti-senescence activity was further validated in two independent models of SHED senescence-replicative exhaustion and doxorubicin-induced stress. Transcriptomic analysis revealed a marked increase in ADCY5 expression following melissic acid treatment. We therefore hypothesized that melissic acid alleviates senescence in SHED by activating the ADCY5/cAMP/CREB axis and downstream autophagy―dependent metabolic remodeling (Stark et al., 2019; Montminy, 1997; Shaywitz and Greenberg, 1999).

Compared with bone marrow-derived MSCs (BMSCs) and adipose-derived MSCs, SHED offer several distinct advantages that make them a highly relevant model for studying stem cell senescence and rejuvenation. First, SHED are readily accessible from naturally exfoliated deciduous teeth, enabling non-invasive collection and avoiding the ethical concerns associated with embryonic stem cells. Second, SHED exhibit higher proliferative activity than BMSCs and dental pulp stem cells (DPSCs)―a characteristic that, while advantageous for expansion, also renders them particularly vulnerable to replicative exhaustion and stress-induced premature senescence. Third, SHED possess strong immunomodulatory properties, with single-cell transcriptomic analyses demonstrating more robust immunomodulatory capacity than BMSCs or umbilical cord-derived MSCs (Kunimatsu et al., 2018). Fourth, SHED display multilineage differentiation potential, including high osteogenic and neurogenic capacity. These unique properties position SHED as an attractive and clinically relevant model for investigating stem cell senescence and for developing strategies to preserve stem cell function.

Importantly, SHED are highly susceptible to senescence during long-term *in vitro* expansion. Studies have shown that SHED undergo senescence following serial passaging to P20, as evidenced by elevated SA-β-Gal positivity, upregulation of p53, p21 and p16Ink4a, decreased proliferation and migration, attenuated differentiation potential, and increased apoptosis. This vulnerability makes SHED an ideal model for screening compounds that can mitigate senescence and restore stem cell function—a key objective of our study. Notably, the senescence pathways activated in SHED differ from those in DPSCs, highlighting the importance of studying senescence specifically in this cell type (Wang et al., 2018; Li et al., 2024).

SHED are a promising postnatal mesenchymal stem cell source for regenerative medicine due to their high proliferative capacity and multilineage differentiation potential (Lamb et al., 2006; Wiley and Campisi, 2016). However, like other adult stem cells, SHED are highly vulnerable to replicative exhaustion and stress-induced premature senescence (Seok et al., 2014; Ning et al., 2023). This progressive loss of function-a process now recognized as stem cell exhaustion-directly compromises their self-renewal and therapeutic utility. Consequently, developing strategies to counteract senescence and restore the regenerative capacity of SHED is of paramount importance for their effective clinical translation.

In the present study, we demonstrate that melissic acid alleviates senescence-associated phenotypes in SHED, suppresses DNA damage signaling, and enhances cAMP/CREB-driven autophagy. Furthermore, it reduces lipid peroxidation, restores mitochondrial membrane potential, and promotes cell-cycle re-entry through activation of the Cyclin D1/Rb axis. Collectively, our findings identify melissic acid as a natural senomorphic candidate for stem cell function restoration and provide a mechanistic link between cAMP―dependent autophagy and the amelioration of stem cell aging, offering a potential strategy to counteract stem cell exhaustion and regenerative decline associated with cellular senescence (Xu et al., 2021).

## 2 Methods

### 2.1 Cell culture and senescence model establishment

Human exfoliated deciduous tooth stem cells (SHED; SALIAI Stem Cell Science and Technology, Guangzhou, China) were cultured in Dulbecco’s modified Eagle’s medium (DMEM; Pricella Biotechnology, Wuhan, China) supplemented with 10% fetal bovine serum (FBS; Winter Song Boye Biotechnology, Beijing, China) and 1% penicillin-streptomycin (Beyotime, China) at 37 °C in a humidified atmosphere containing 5% CO_2_. Cells at passage 6 (P6) were used as the low-passage control throughout this study. Replicative senescence was induced by continuous serial passaging until passages 15–20 (P15–P20), as previously described for stem cell aging models (Gronthos et al., 2000; Campisi and d'Adda di Fagagna, 2007). Stress-induced premature senescence was established by exposing low-passage SHED to 0.2 μM doxorubicin (ADR; Aladdin, China) for 24 h, followed by a 24 h recovery period in drug-free medium. Senescence phenotypes were verified by SA-β-Gal staining and the expression of canonical senescence markers before downstream experiments.

### 2.2 High-throughput phenotypic screening

To identify phytochemicals and other natural compounds with anti-senescence activity, a robot-assisted high-throughput screen was performed in replicatively senescent SHED (P20). A library of 800 natural small molecules (each at 10 μM) was added using an automated liquid handling system. After 48 h of incubation, SA-β-Gal staining was performed to assess anti-senescence efficacy. Compounds that reduced SA-β-Gal positivity by ≥50% compared to vehicle control were considered primary hits. The five most effective candidates were selected for further validation, among which melissic acid exhibited the most favorable efficacy-safety profile.

### 2.3 Melissic acid preparation and treatment

Melissic acid (triacontanoic acid; purity ≥98%; TargetMol Chemicals, China) was dissolved in dimethyl sulfoxide (DMSO; MedChemExpress, USA) to prepare a 10 mM stock solution and stored at −20 °C in aliquots protected from light. Working solutions were freshly prepared in complete medium immediately before use. Senescent SHED (both replicative and ADR-induced) were treated with melissic acid at final concentrations of 5, 10, or 20 μM for 48 h. Vehicle controls received an equivalent volume of DMSO (final concentration ≤0.1%, v/v).

### 2.4 Cell viability assay

Cell viability was determined using a Cell Counting Kit-8 (CCK-8; Beyotime, China) according to the manufacturer’s instructions. SHED were seeded in 96-well plates at 3,000–5,000 cells per well and allowed to attach overnight. Cells were then treated with melissic acid (0, 5, 10, or 20 μM) for 48 h. Subsequently, 10 μL of CCK-8 reagent was added to each well containing 100 μL of culture medium, and the plates were incubated at 37 °C for 2–4 h. Absorbance at 450 nm was measured using a microplate reader (BioTek, United States). Cell viability was expressed as a percentage of the untreated control.

### 2.5 Senescence-associated β-galactosidase (SA-β-Gal) staining

SA-β-Gal staining was performed using a Senescence β-Galactosidase Staining Kit (Beyotime, China) following the manufacturer’s protocol. Briefly, cells were washed twice with PBS, fixed with fixation solution for 15 min at room temperature, and then incubated with SA-β-Gal staining solution at 37 °C overnight in a CO_2_-free incubator. Blue-stained cells were imaged under an inverted microscope (Nikon ECLIPSE Si, Japan). At least three randomly selected fields per sample were analyzed, and the percentage of SA-β-Gal-positive cells was quantified using ImageJ software (NIH, United States).

### 2.6 Western blot analysis

Total protein was extracted using RIPA lysis buffer (Beyotime, China) supplemented with 1 mM phenylmethylsulfonyl fluoride (PMSF) and a phosphatase inhibitor cocktail. Protein concentration was measured using a BCA Protein Assay Kit (Beyotime, China). Equal amounts of protein (20–40 μg per lane) were separated by 10% or 12% SDS-PAGE and transferred to polyvinylidene difluoride (PVDF) membranes (Bio-Rad, USA). Membranes were blocked with 5% non-fat milk or 5% bovine serum albumin (BSA) in TBST for 1 h at room temperature and then incubated overnight at 4 °C with primary antibodies against p16 (Abclonal, China), p21 (MedChemExpress, United States), p53 (Beyotime, China), γ-H2AX (MedChemExpress, United States), p-CREB (Ser133) (Selleck Chemicals, United States), CREB (Selleck Chemicals, United States), p-Rb (Ser780) (Selleck Chemicals, United States), Rb (Selleck Chemicals, United States), Cyclin D1 (Selleck Chemicals, United States), p62/SQSTM1 (Immunoway Biotechnology Company, United States), Beclin-1 (Selleck Chemicals, United States), LC3B (Selleck Chemicals, United States), and GAPDH (Beyotime, China) (as a loading control). After washing, membranes were incubated with horseradish peroxidase-conjugated secondary antibodies for 1 h at room temperature. Protein bands were visualized using enhanced chemiluminescence (ECL; Abbkine Scientific, China) and captured with a ChemiDoc imaging system (ShenHua Technology, China). Densitometric analysis was performed using ImageJ, and target protein levels were normalized to GAPDH or to total protein for phosphorylated targets.

### 2.7 Immunofluorescence staining

Cells grown on culture dishes were fixed with 4% paraformaldehyde for 15 min, permeabilized with PBS containing 0.2% Triton X-100 for 10 min, and blocked with 5% BSA for 1 h at room temperature. They were then incubated overnight at 4 °C with primary antibodies against p16, p21, or γ-H2AX. After washing, cells were incubated in the dark with Alexa Fluor 488- or 594-labeled secondary antibodies for 1 h. Nuclei were counterstained with DAPI contained in the mounting medium. Fluorescence images were acquired using a high-content microscope under identical conditions. Quantitative analysis of average fluorescence intensity was performed using ImageJ on at least 50 cells per group from three independent experiments.

### 2.8 RNA sequencing and bioinformatic analysis

Total RNA was extracted from vehicle-treated and melissic acid-treated (10 μM, 48 h) senescent SHED using TRIzol reagent (Solarbio Science and Technology, China). RNA concentration and purity were assessed with a NanoDrop 2000 spectrophotometer (Thermo Fisher, United States), and RNA integrity was confirmed using an Agilent 2100 Bioanalyzer (Agilent Technologies, United States). Only samples with an RNA integrity number (RIN) ≥ 7.0 were used for library preparation. Paired-end sequencing (150 bp) was performed on an Illumina NovaSeq 6000 platform. Raw reads were trimmed using Trimmomatic and aligned to the human reference genome (GRCh38) using HISAT2. Transcript abundance was quantified as fragments per kilobase of transcript per million mapped reads (FPKM) using StringTie. This RNA-seq experiment was performed with two biological replicates per condition (n = 2) and was designed as a discovery/screening tool to identify candidate genes with consistent fold changes, rather than as a confirmatory test. Differential expression analysis was performed using DESeq2, and genes with |log_2_(fold change)| > 1 and nominal P < 0.05 were considered as candidates for further validation (Ziegenhain et al., 2017; Lamarre et al., 2018; Luo et al., 2025). Heatmaps were generated using the pheatmap package in R with Z-score normalization. All candidate genes identified from this screening were subsequently validated by quantitative real-time PCR (qPCR) with three independent biological replicates (n = 3), and the mechanistic conclusions of this study are based on these qPCR-validated data.

### 2.9 Quantitative real-time PCR (qPCR)

Total RNA was reverse-transcribed into cDNA using the PrimeScript RT Reagent Kit with gDNA Eraser (Foreverstar Biotechnology, China). qPCR was performed on a QuantStudio 6Pro Real-Time PCR System (Thermo Fisher, United States). The cycling conditions were: 95 °C for 30 s, followed by 40 cycles of 95 °C for 5 s and 60 °C for 34 s. Melting curve analysis confirmed primer specificity. Relative gene expression was calculated using the 2^−ΔΔCt^ method, with GAPDH as the internal reference (Livak and Schmittgen, 2001). Primers for ADCY5, AMDHD1, EPHA3, PLIN4, LAMP3, MYOM2, p53, Cyclin D1, and GAPDH were synthesized by General Bio (Anhui) Co., Ltd., China, and are listed in Supplementary Table S1.

### 2.10 Cell cycle analysis

Cell cycle distribution was analyzed by propidium iodide (PI) staining and flow cytometry. After treatment, cells were harvested, washed with cold PBS, and fixed in 70% ice-cold ethanol overnight at −20 °C. Fixed cells were washed and incubated with PI/RNase staining buffer (Beyotime, China) for 30 min at 37 °C in the dark. DNA content was analyzed using a Beckman CytoFLEX flow cytometer (Beckman Coulter, United States), and at least 10,000 events were collected per sample. Cell cycle distribution was calculated using FlowJo software (version 10.0, Tree Star, United States) (Sharpless and Sherr, 2015).

### 2.11 Assessment of mitochondrial membrane potential (ΔΨm)

Mitochondrial membrane potential was evaluated using a JC-1 Mitochondrial Membrane Potential Detection Kit (Beyotime, China) (Livak and Schmittgen, 2001). After treatment, cells were incubated with JC-1 working solution for 20 min at 37 °C in the dark, washed twice with JC-1 staining buffer, and immediately observed under a fluorescence microscope. The red/green fluorescence ratio was used as an indicator of ΔΨm.

### 2.12 Lipid peroxidation assay

Lipid peroxidation was assessed using a BODIPY 581/591 C11 Lipid Peroxidation Sensor (Beyotime, China) (Krishan, 1975). Cells were incubated with 5 μM BODIPY 581/591 C11 in serum-free medium for 30 min at 37 °C in the dark, washed with PBS, and immediately imaged using a fluorescence microscope. Oxidized probe fluorescence was detected as a shift from red to green, and the red/green fluorescence ratio was calculated as a readout of lipid peroxidation.

### 2.13 Statistical analysis

All experiments were independently repeated at least three times. Data are presented as mean ± standard deviation (SD). Statistical analyses were performed using GraphPad Prism 9.0 (GraphPad Software, United States). Comparisons between two groups were analyzed using an unpaired two-tailed Student’s t-test, while comparisons among multiple groups were performed using one-way analysis of variance (ANOVA) followed by Tukey’s *post hoc* test. A P value < 0.05 was considered statistically significant.

## 3 Results

### 3.1 High-throughput phenotypic screening identifies melissic acid as a candidate anti-senescence agent in senescent SHED

To identify phytochemicals and other natural compounds with anti-senescence activity, we established two complementary models of SHED senescence: replicative senescence induced by serial passaging (P20) and stress-induced premature senescence triggered by doxorubicin (also termed ADR). A phenotypic screen of 800 natural compounds was performed in replicatively senescent SHED using a robot-assisted high-throughput platform. Following 48 h of compound treatment, anti-senescence efficacy was assessed by SA-β-Gal staining (Figure 1A). Among the 792 compounds successfully evaluated, five compounds-Larixyl acetate, 2′-Deoxyguanosine, α-Terpinene, Vanillin, and melissic acid-exhibited marked anti-senescence activity as shown in the scatter plot distribution (Figure 1B). The anti-senescence effect of these five candidates was confirmed by SA-β-Gal staining in high-passage SHED, with representative images and quantitative analyses presented in Figures 1C,D, respectively. To evaluate potential cytotoxicity, CCK-8 assays were performed on both low-passage (P6) and high-passage (P20) SHED treated with increasing concentrations of each compound. Among the five hits, melissic acid demonstrated the most favorable profile: it effectively reduced senescence-associated β-galactosidase activity while causing minimal cytotoxicity (Figure 1E). We therefore selected melissic acid for further mechanistic investigation. A dose-response CCK-8 assay in low-passage SHED, replicatively senescent SHED, and doxorubicin-induced senescent SHED confirmed that melissic acid (5–20 μM) exhibited negligible cytotoxicity in low-passage cells and only modest toxicity (70–80% viability) at the highest concentration (20 μM) in senescent cells, indicating a satisfactory safety window (Figure 1F). Collectively, these data identify melissic acid as a promising senomorphic candidate for mitigating SHED senescence. To determine whether the modest reduction in cell viability observed in CCK-8 assays reflected apoptotic cell death, we performed TUNEL staining in senescent SHED treated with melissic acid (10 μM, 48 h). As shown in Supplementary Figure S1, TUNEL-positive cells were rarely detected across all groups, and there were no obvious differences between vehicle-treated and melissic acid-treated senescent cells in either the replicative senescence (P20) or doxorubicin-induced (ADR) models. These results confirm that melissic acid does not induce apoptosis in senescent SHED, further supporting its classification as a senomorphic rather than senolytic agent.

**FIGURE 1:**
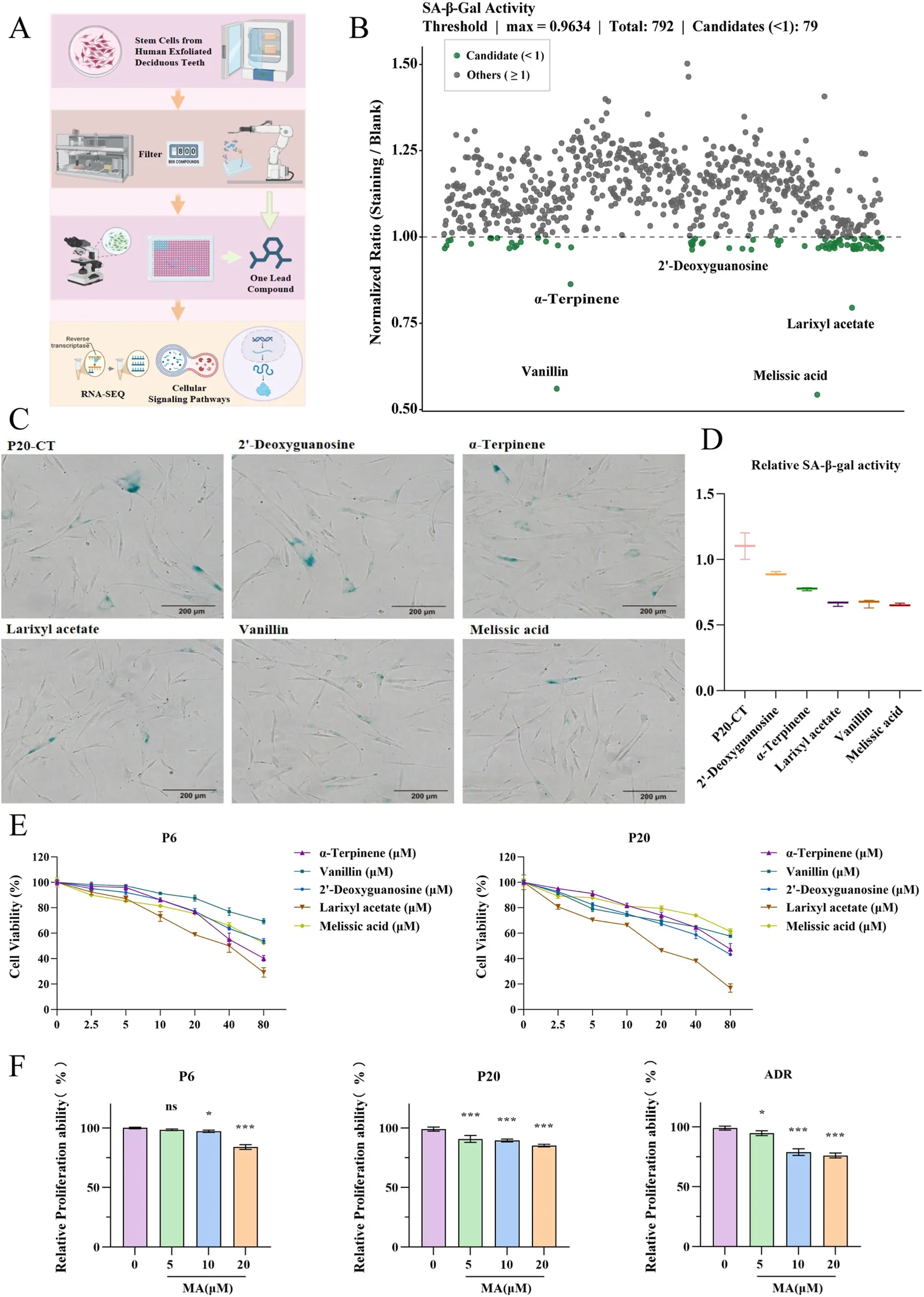
High-throughput phenotypic screening identifies melissic acid as a candidate anti-senescence compound in replicatively senescent SHED. **(A)** Schematic workflow of the robot-assisted high-throughput small-molecule screening in passage 20 (P20) replicatively senescent SHED. Images were created with BioRender.com under permission. **(B)** Scatter plot showing the screening results for 792 natural compounds (10 μM, 48 h). The dashed line at 0.9634 marks the cutoff for initial data quality filtering (normalized ratio < 1); 79 compounds fell below this threshold and were considered as having potential anti-senescence activity. Five compounds (highlighted) were selected for further validation based on their most pronounced and reproducible effects. **(C)** Representative bright-field images of SA-β-Gal staining in high-passage SHED treated with each of the five candidate compounds (10 μM, 48 h). Scale bar, 200 μm. **(D)** Quantification of SA-β-Gal-positive cells after treatment with the five candidate compounds. **(E)** CCK-8 assay showing the dose-dependent effects of the five candidate compounds (0–80 μM) on viability of low-passage (P6) and high-passage (P20) SHED. **(F)** CCK-8 analysis of low-passage SHED, replicatively senescent SHED, and doxorubicin-induced senescent SHED treated with melissic acid at the indicated concentrations (5, 10, 20 μM), demonstrating minimal cytotoxicity in low-passage cells and acceptable safety in senescent cells. Data are presented as mean ± SD from at least three independent experiments. ns, not significant; *p < 0.05, **p < 0.01, ***p < 0.001 versus the respective control group.

### 3.2 Melissic acid suppresses classical senescence markers and DNA damage signals in senescent SHED

To establish a reliable stress-induced premature senescence model, we treated low-passage (P5) SHED with increasing concentrations of doxorubicin (0–2 μM) for 24 h, followed by a 24 h recovery in normal medium. SA-β-Gal staining revealed that 0.2 μM doxorubicin induced the most pronounced senescence phenotype, as evidenced by the highest percentage of SA-β-Gal-positive cells (Figures 2A,B). This concentration was therefore selected for subsequent experiments. We next evaluated the anti-senescence effect of melissic acid in both replicatively senescent (P20) and doxorubicin-induced senescent SHED. The ADR-induced senescence model was further validated by Western blot analysis, which confirmed upregulation of p53, p21, and p16 in ADR-treated SHED compared with low-passage controls (Figure 3A). As shown in Figures 2C,D, treatment with melissic acid at 5–20 μM for 48 h significantly reduced SA-β-Gal positivity in a dose-dependent manner, with 10 μM already achieving a marked reduction in both senescence models. To assess whether melissic acid attenuates DNA damage signaling, we performed immunofluorescence staining for γ-H2AX. Senescent SHED exhibited strong γ-H2AX foci, whereas melissic acid treatment (5–20 μM) substantially decreased γ-H2AX fluorescence intensity in both replicatively senescent and doxorubicin-induced senescent cells, with the most pronounced effect observed at 10 μM and 20 μM (Figures 2E,F). Collectively, these results demonstrate that melissic acid alleviates senescence-associated phenotypes and suppresses DNA damage responses in SHED.

**FIGURE 2:**
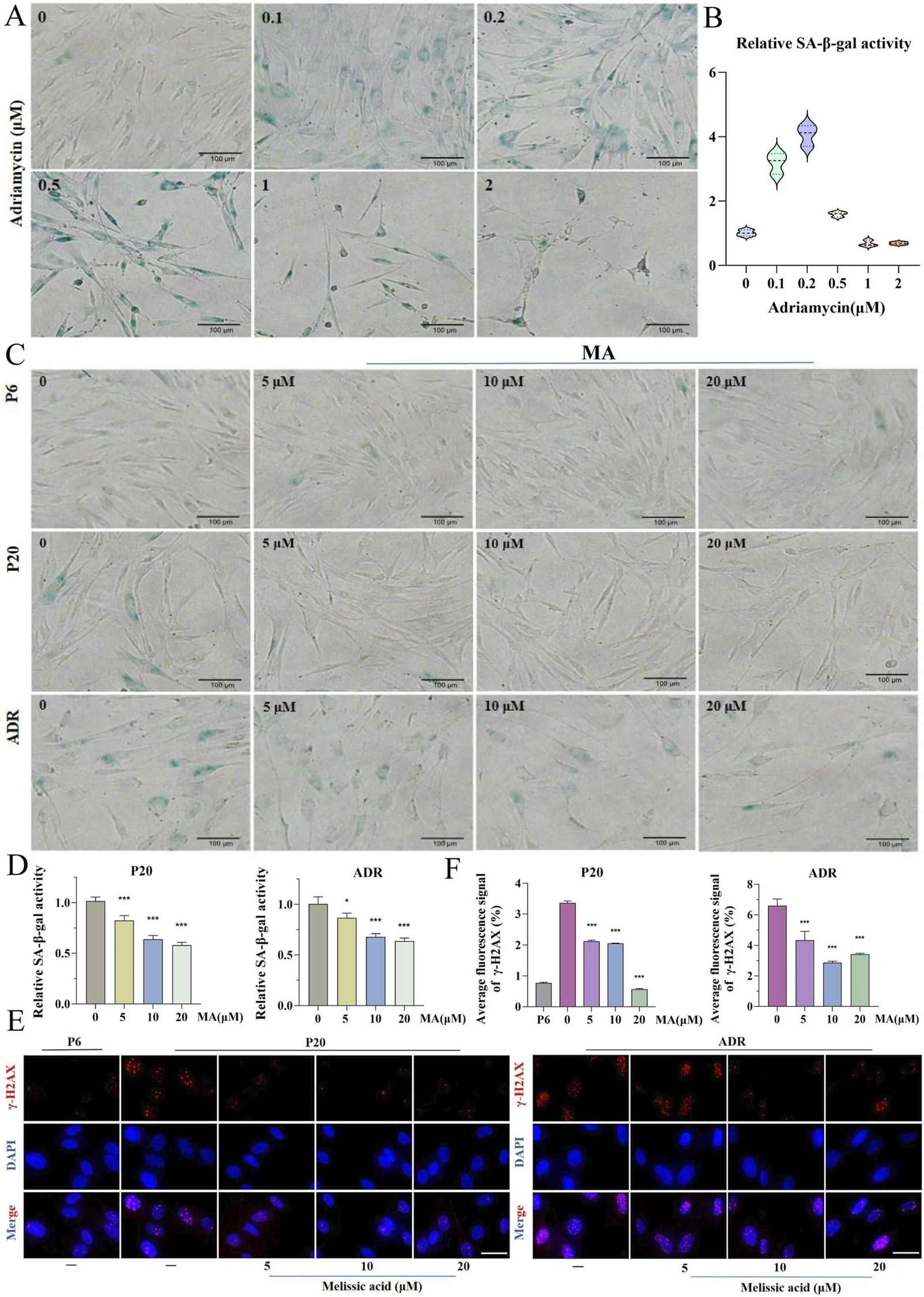
Melissic acid alleviates senescence phenotypes in replicative and doxorubicin-induced SHED models. **(A)** Representative bright-field images of SA-β-Gal staining in low-passage (P5) SHED treated with increasing concentrations of doxorubicin (0–2 μM) for 24 h followed by 24 h recovery, showing establishment of stress-induced premature senescence. Scale bar, 100 μm. **(B)** Quantification of SA-β-Gal-positive cells from **(A)**; 0.2 μM doxorubicin was selected for subsequent experiments. **(C)** Representative SA-β-Gal staining images of low-passage SHED (P6), replicatively senescent SHED (P20), doxorubicin-induced senescent SHED, and melissic acid-treated groups at the indicated concentrations (5, 10, 20 μM). Scale bar, 100 μm. **(D)** Quantification of SA-β-Gal-positive cells in replicatively senescent and doxorubicin-induced senescent SHED after melissic acid treatment, showing significant reduction starting at 10 μM. **(E)** Representative immunofluorescence images of γ-H2AX staining in low-passage (P6), replicatively senescent (P20), and melissic acid-treated (5, 10, 20 μM) SHED. Scale bar, 30 μm. **(F)** Quantification of γ-H2AX fluorescence intensity from **(E)**, showing reduction after melissic acid treatment at all tested concentrations. Data are presented as mean ± SD from three independent experiments. Statistical significance was determined relative to the respective vehicle-treated control group. *p < 0.05, **p < 0.01, ***p < 0.001.

**FIGURE 3:**
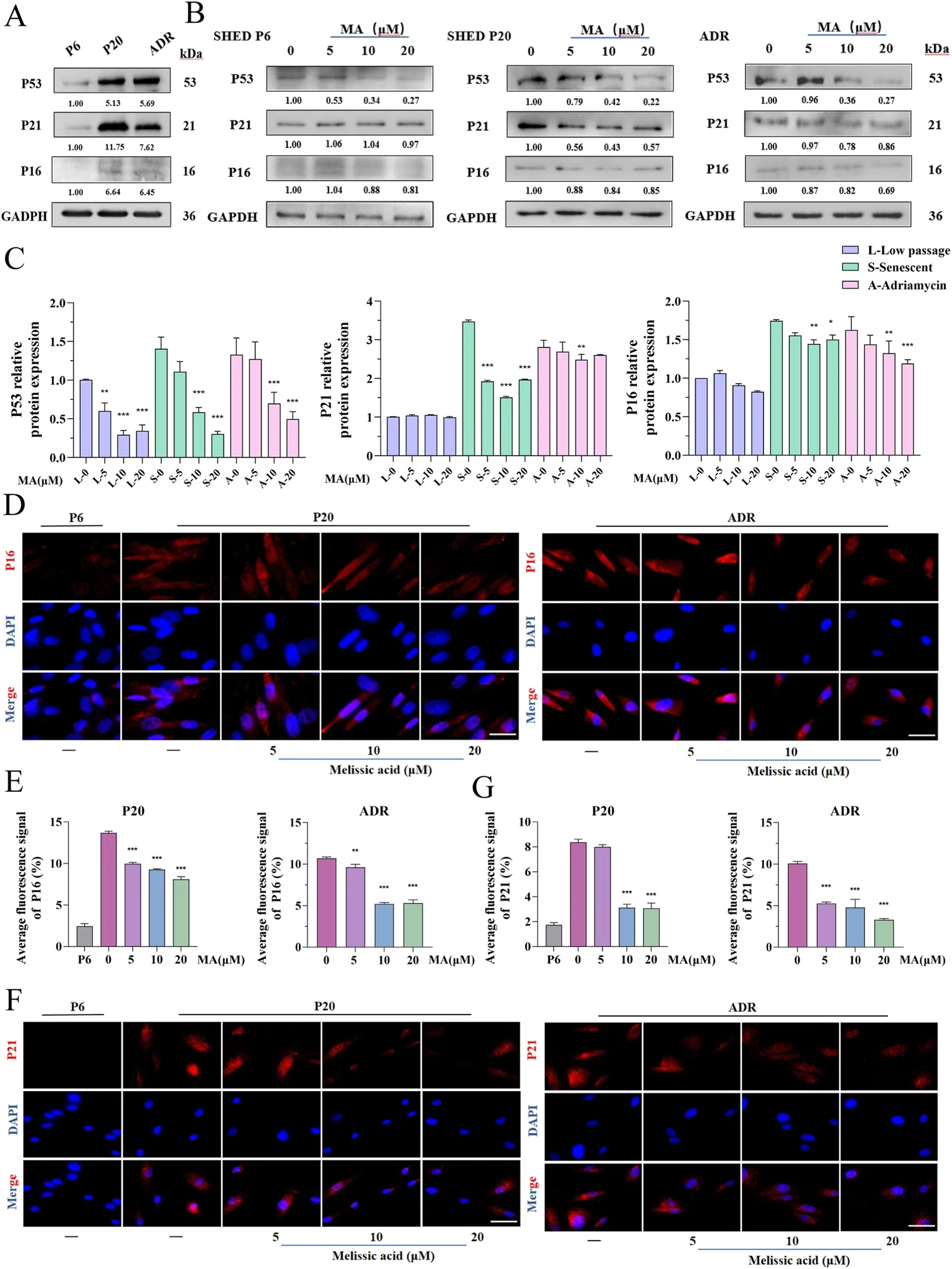
Melissic acid suppresses canonical senescence markers p53, p21, and p16 in senescent SHED. **(A)** Representative Western blot images of p53, p21, and p16 in low-passage (P6), replicatively senescent (P20), and doxorubicin-induced senescent (ADR) SHED, showing marked upregulation of these senescence markers in both senescent models. **(B)** Representative Western blot images of p53, p21, and p16 in replicatively senescent and doxorubicin-induced senescent SHED treated with melissic acid at the indicated concentrations (5, 10, 20 μM) for 48 h, showing dose-dependent reductions. **(C)** Densitometric quantification of p53, p21, and p16 protein expression from **(B)**, normalized to loading controls. **(D)** Representative immunofluorescence images of p16 staining in low-passage, senescent, and melissic acid-treated (10 μM) SHED. Scale bar, 30 μm. **(E)** Quantification of p16 fluorescence intensity from **(D)**, demonstrating significant reduction after melissic acid treatment. **(F)** Representative immunofluorescence images of p21 staining in the indicated groups. Scale bar, 30 μm. **(G)** Quantification of p21 fluorescence intensity from **(F)**. Data are presented as mean ± SD from at least three independent experiments. ns, not significant; *p < 0.05, **p < 0.01, ***p < 0.001 versus the respective control group.

### 3.3 Melissic acid reduces expression of canonical senescence regulators p53, p21, and p16 in senescent SHED

To further characterize the anti-senescence effect of melissic acid, we examined the expression of key cell-cycle arrest and senescence-associated proteins. Western blot analysis revealed that p53, p21, and p16 were markedly upregulated in both replicatively senescent (P20) and doxorubicin-induced senescent SHED compared with low-passage controls, confirming successful establishment of both senescence models (Figure 3A). Treatment with melissic acid at 5–20 μM for 48 h dose-dependently reduced the abundance of p53, p21, and p16 in senescent SHED (Figure 3B). Densitometric quantification of the Western blot bands confirmed the significant downregulation of these proteins after melissic acid treatment (Figure 3C). Immunofluorescence staining further supported these findings: p16 fluorescence intensity was substantially decreased in melissic acid-treated senescent SHED compared with untreated senescent controls (Figures 3D,E). Similarly, p21 immunofluorescence signals were markedly attenuated following melissic acid treatment (Figures 3F,G). Collectively, these results demonstrate that melissic acid suppresses canonical senescence-associated proteins in senescent SHED at both the protein expression level and cellular localization.

### 3.4 Transcriptomic profiling identifies ADCY5 as a key target of melissic acid and qPCR validation confirms its modulation

To uncover the molecular mechanisms underlying the anti-senescence effect of melissic acid, we performed RNA sequencing on low-passage (P6), replicatively senescent (P20), and melissic acid-treated (10 μM, 48 h) senescent SHED. Comparison between low-passage and senescent SHED revealed numerous differentially expressed genes, as shown in the volcano plot (Figure 4A). We note that the genes highlighted in this plot―PLIN4, AMDHD1, MYOM2, LAMP3―were not selected based on their significance in the senescence comparison. Rather, they are highlighted because they exhibited pronounced and consistent expression changes upon melissic acid treatment (Figures 4B,D). This pattern indicates that melissic acid does not simply reverse the global senescence transcriptome, but rather selectively modulates specific pathways. In contrast, comparison between senescent SHED and melissic acid-treated senescent SHED identified six genes with pronounced and consistent expression changes: ADCY5, AMDHD1, and EPHA3 were significantly upregulated, whereas PLIN4, LAMP3, and MYOM2 were markedly downregulated (Figure 4B). Functional pathway analysis using a heatmap of genes associated with cAMP signaling, autophagy, and cell-cycle regulation showed coordinated alterations across the three groups, indicating that melissic acid broadly modulates these pathways (Figure 4C). Given the exploratory nature of the RNA-seq dataset, we performed quantitative real-time PCR (qPCR) with three independent biological replicates (n = 3) to validate the six candidate genes together with p53 and Cyclin D1. As shown in Figure 4D, the expression of ADCY5, AMDHD1, and EPHA3 was significantly decreased in senescent SHED compared with low-passage controls, and melissic acid treatment restored their expression to levels approaching those of low-passage cells. Conversely, PLIN4, LAMP3, and MYOM2 were upregulated in senescent SHED and were significantly suppressed by melissic acid. Additionally, p53, a canonical senescence marker, was elevated in senescent SHED and reduced upon melissic acid treatment, whereas Cyclin D1, a key cell-cycle activator, showed the opposite trend: downregulation in senescent cells and restoration after melissic acid treatment. These qPCR results are consistent with the RNA-seq data and further support the anti-senescence role of melissic acid through upregulation of ADCY5 and modulation of downstream pathways. To determine whether these molecular changes translate into functional recovery of stem cell potency, we performed colony-forming efficiency (CFE) assays in senescent SHED treated with melissic acid (10 μM, 48 h). As shown in Figure 4E, the clonogenic capacity of senescent SHED (P20 and ADR) was significantly reduced compared with low-passage controls (P6). Notably, melissic acid treatment markedly increased both the number and size of colonies formed by senescent SHED, restoring clonogenic capacity to approximately 60–70% of low-passage levels (p < 0.01). These results demonstrate that the anti-senescence effects of melissic acid at the molecular level translate into functional recovery of stem cell potency. Given the prominent and consistent upregulation of ADCY5, we hypothesized that melissic acid may exert its anti-senescence effect through activation of the ADCY5/cAMP/CREB signaling axis.

**FIGURE 4:**
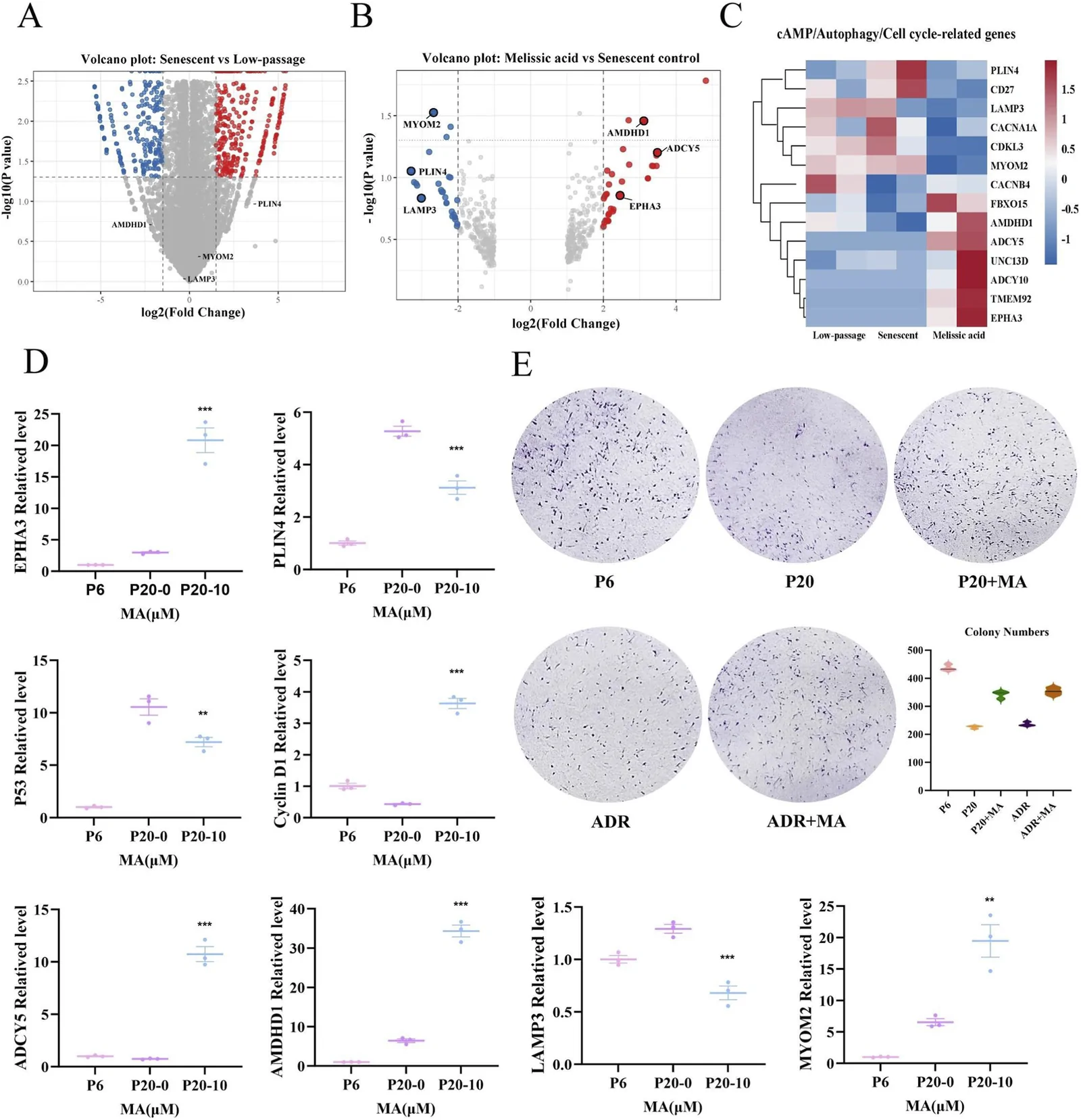
Transcriptomic profiling identifies melissic acid-regulated genes and signaling pathways in senescent SHED. **(A)** Volcano plot showing differentially expressed genes between low-passage (P6) and replicatively senescent (P20) SHED from an exploratory RNA-seq analysis (n = 2 per group). The highlighted genes (PLIN4, AMDHD1, MYOM2, LAMP3) were marked not for their significance in the senescence comparison, but because they exhibited pronounced changes upon melissic acid treatment [see **(B)** and **(D)**]. **(B)** Volcano plot showing differentially expressed genes between senescent SHED and melissic acid-treated (10 μM, 48 h) senescent SHED. Six genes―ADCY5, AMDHD1, EPHA3 (upregulated), PLIN4, LAMP3, MYOM2 (downregulated)―displayed pronounced changes. **(C)** Heatmap of genes associated with cAMP signaling, autophagy, and cell-cycle-related pathways across low-passage SHED, senescent SHED, and melissic acid-treated senescent SHED. **(D)** qPCR validation of the six candidate genes together with p53 and Cyclin D1 in low-passage, senescent, and melissic acid-treated senescent SHED. **(E)** Melissic acid restores clonogenic capacity of senescent SHED. Representative images of crystal violet-stained colonies formed by low-passage (P6), replicatively senescent (P20), doxorubicin-induced senescent (ADR), and melissic acid-treated (10 μM, 48 h) senescent SHED. Cells were seeded at low density (400 cells/well in 6-well plates) and cultured for 10–14 days after treatment. Data are presented as mean ± SD from three independent experiments. ns, not significant; *p < 0.05, **p < 0.01, ***p < 0.001 versus the respective control group.

### 3.5 Melissic acid activates cAMP/CREB signaling, relieves G0/G1 arrest, and promotes cell-cycle re-entry in senescent SHED

To test whether the cAMP/CREB pathway is involved in melissic acid-mediated senomorphic modulation, we examined CREB phosphorylation at Ser133. Western blot analysis showed that melissic acid (5–20 μM, 48 h) significantly increased p-CREB (Ser133) levels in both replicatively senescent and doxorubicin-induced senescent SHED, whereas total CREB expression remained unchanged, indicating specific activation of the cAMP/CREB signaling pathway (Figures 5C,D). To directly confirm that the transcriptional upregulation of ADCY5 translates into increased cAMP production, we measured intracellular cAMP levels using a competitive ELISA assay. As shown in Supplementary Figure S2, cAMP levels were significantly lower in senescent SHED (P20 and ADR) compared with low-passage (P6) controls. Notably, melissic acid treatment (10 μM, 48 h) significantly elevated cAMP levels in both replicatively senescent (from approximately 10–12.5 ng/mL, ***p < 0.001) and ADR-induced senescent (from approximately 6–10.5 ng/mL, ***p < 0.001) SHED. To further validate that ADCY5 is required for melissic acid-induced anti-senescence effects, we performed pharmacological inhibition experiments using SQ22536, an adenylate cyclase inhibitor. Senescent SHED were pretreated with SQ22536 (50 μM, 1 h) prior to co-treatment with melissic acid (10 μM) for 48 h. As shown in Supplementary Figure S3, SA-β-Gal staining demonstrated that melissic acid markedly reduced senescence-associated β-galactosidase activity in P20 model, whereas SQ22536 pretreatment effectively reversed this effect. Consistent results were observed in the ADR model (Supplementary Figure S4). Western blot analysis (Supplementary Figure S4) showed that SQ22536 pretreatment blocked melissic acid-induced p-CREB elevation and p21 suppression, confirming that ADCY5 is required for the anti-senescence activity of melissic acid in SHED. We next examined whether melissic acid could reactivate proliferative signaling in senescent SHED. Flow cytometric analysis revealed that low-passage SHED exhibited a normal cell-cycle distribution, while replicatively senescent SHED showed a significantly higher proportion of cells in the G0/G1 phase. Treatment with melissic acid at 10 μM for 48 h substantially decreased the G0/G1 fraction and increased the percentages of S and G2/M phase cells in senescent SHED, whereas low-passage cells showed no significant changes (Figures 5A,B). Consistently, Western blot analysis demonstrated that melissic acid dose-dependently increased the expression of Cyclin D1 and enhanced phosphorylation of Rb at Ser780, while total Rb levels remained unchanged (Figures 5C,D). These data provide direct biochemical evidence that melissic acid activates the ADCY5/cAMP signaling axis. These findings indicate that melissic acid alleviates senescence-associated G0/G1 arrest by reactivating the Cyclin D1/Rb axis, thereby promoting cell-cycle re-entry in senescent SHED.

**FIGURE 5:**
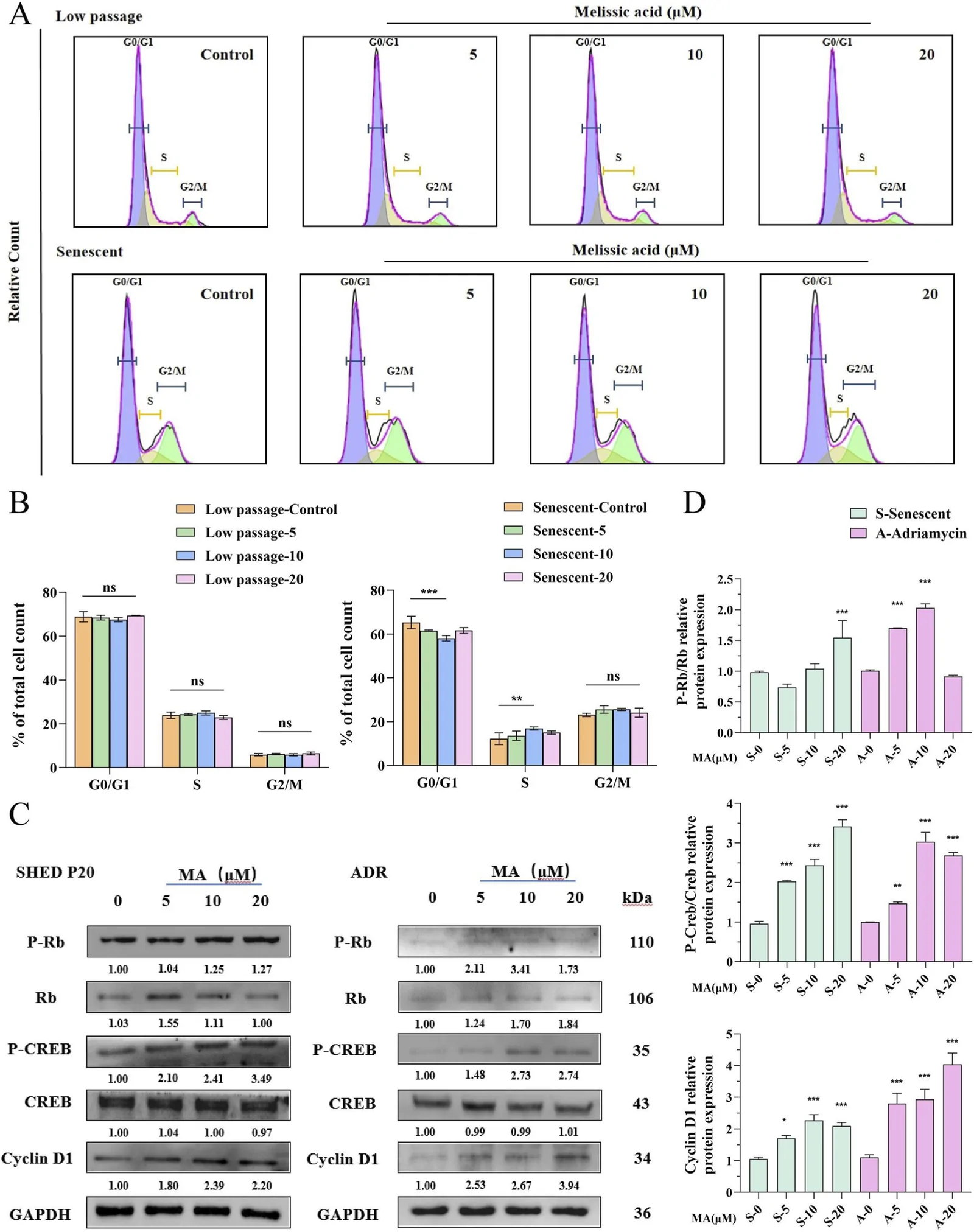
Melissic acid activates cAMP/CREB signaling and promotes cell-cycle re-entry in senescent SHED. **(A)** Representative flow cytometry plots of cell-cycle distribution in low-passage SHED (P6), replicatively senescent SHED (P20), and melissic acid-treated (5, 10, 20 μM, 48 h) senescent SHED. **(B)** Quantification of the percentage of cells in G0/G1, S, and G2/M phases from **(A)**. Low-passage cells showed no significant changes; senescent cells treated with melissic acid exhibited decreased G0/G1 and increased S and G2/M phase fractions in a dose-dependent manner, with the most pronounced effect at 10 μM. **(C)** Representative Western blot images of p-CREB (Ser133), CREB, p-Rb (Ser780), Rb, and Cyclin D1 in replicatively senescent (P20), and doxorubicin-induced senescent (ADR) SHED, with or without melissic acid treatment (5, 10, 20 μM, 48 h). **(D)** Densitometric quantification of p-CREB, p-Rb, and Cyclin D1 expression from **(C)**, normalized to total CREB, total Rb, or loading controls, respectively. Data are presented as mean ± SD from at least three independent experiments. ns, not significant; *p < 0.05, **p < 0.01, ***p < 0.001 versus the respective control group.

### 3.6 Melissic acid enhances autophagy, restores mitochondrial membrane potential, and reduces lipid peroxidation in senescent SHED

To determine whether melissic acid activates autophagy in senescent SHED, we examined the expression of key autophagy-related proteins. Western blot analysis revealed that melissic acid (5–20 μM, 48 h) dose-dependently decreased p62/SQSTM1 expression and increased Beclin-1 levels in both replicatively senescent and doxorubicin-induced senescent SHED (Figures 6A,B), indicating enhanced autophagic activity. To further confirm that melissic acid enhances autophagic flux, we examined LC3B expression and performed chloroquine (CQ) co-treatment experiments. As shown in Figure 6A, melissic acid treatment (10 μM, 48 h) increased LC3B levels in both P20 and ADR models. Chloroquine (50 μM) was added during the final 6 h of treatment to block lysosomal degradation. As shown in Supplementary Figure S5, chloroquine co-treatment further accumulated LC3B in melissic acid-treated cells compared with melissic acid alone (LC3B levels: 1.60 vs. 1.12 in P20+MA + CQ vs. P20 + MA alone). These results confirm that melissic acid enhances complete autophagic flux rather than merely blocking autophagosome degradation. Because autophagy is closely linked to mitochondrial quality control, we next evaluated mitochondrial membrane potential using JC-1 staining. Senescent SHED exhibited a decreased red/green fluorescence ratio, reflecting mitochondrial depolarization, whereas melissic acid treatment significantly increased the red/green ratio, indicating restoration of mitochondrial membrane potential (Figures 6C,D). Furthermore, we assessed lipid peroxidation, a marker of oxidative damage, using BODIPY 581/591 C11 staining. Senescent SHED showed a reduced red/green fluorescence ratio compared with low-passage controls, indicating elevated lipid peroxidation (Drummen et al., 2002). Melissic acid treatment markedly increased the red/green ratio, suggesting attenuation of lipid peroxidation (Figures 6E,F). Taken together, these results demonstrate that melissic acid enhances autophagy, improves mitochondrial function, and reduces oxidative damage in senescent SHED.

**FIGURE 6:**
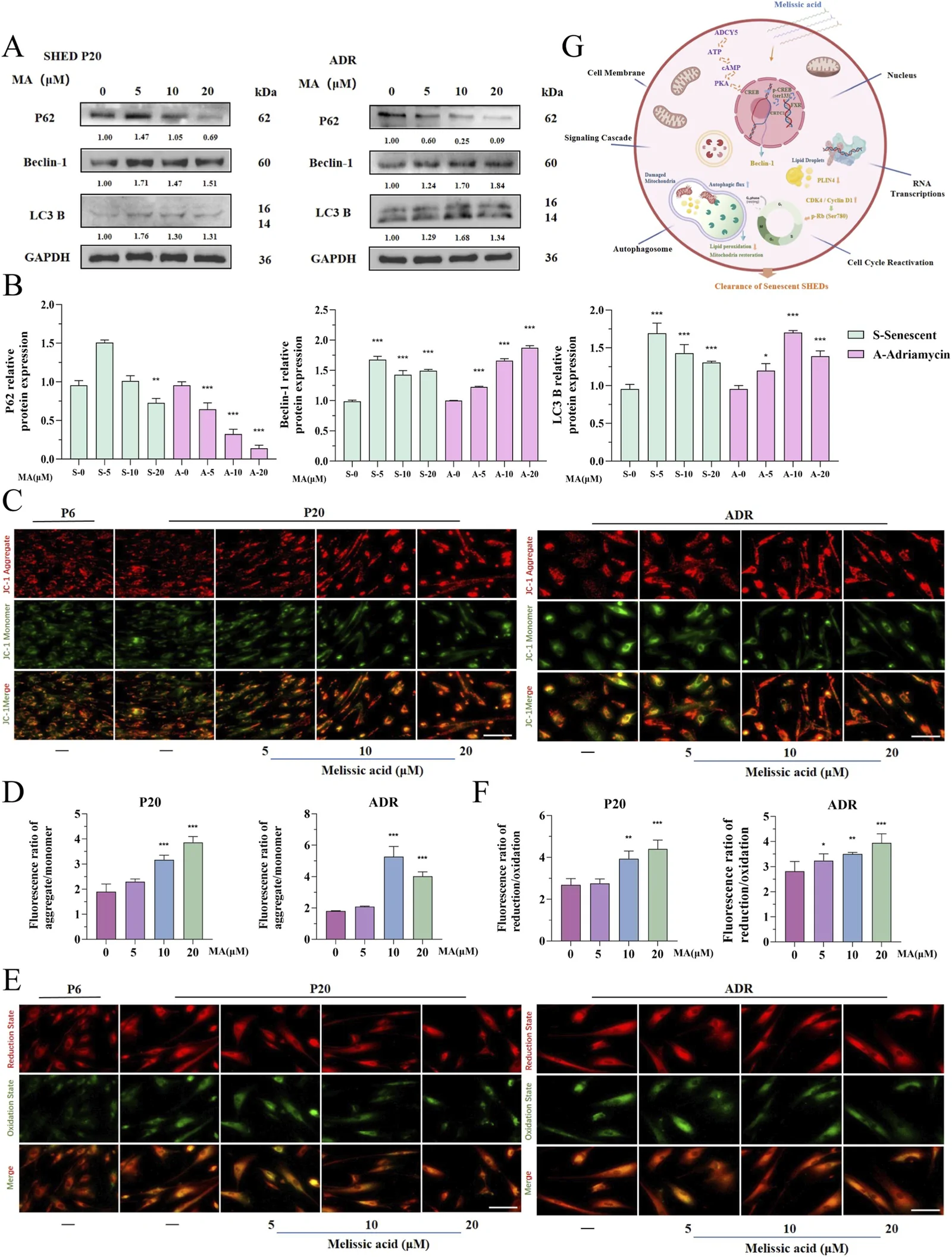
Melissic acid enhances autophagy, restores mitochondrial membrane potential, reduces lipid peroxidation, and alleviates SHED senescence. **(A)** Representative Western blot images of p62/SQSTM1 and Beclin-1 in replicatively senescent (P20) and doxorubicin-induced senescent (ADR) SHED treated with melissic acid (5, 10, 20 μM, 48 h). **(B)** Densitometric quantification of p62 and Beclin-1 expression from **(A)**, normalized to loading controls. **(C)** Representative JC-1 staining images showing mitochondrial membrane potential (ΔΨm) in low-passage (P6), replicatively senescent (P20), doxorubicin-induced senescent (ADR), and melissic acid-treated (5, 10, 20 μM) senescent SHED. Scale bar, 50 μm. **(D)** Quantification of the JC-1 red/green fluorescence ratio from **(C)**, demonstrating restoration of mitochondrial membrane potential after melissic acid treatment. **(E)** Representative BODIPY 581/591 C11 staining images showing lipid peroxidation in the indicated groups. Scale bar, 50 μm. **(F)** Quantification of lipid peroxidation levels based on the red/green fluorescence ratio from **(E)**, showing reduction after melissic acid treatment. **(G)** Schematic illustration of the proposed mechanism by which melissic acid alleviates SHED senescence. Melissic acid upregulates ADCY5, leading to increased cAMP production and CREB phosphorylation (Ser133), which in turn promotes autophagic flux and restores mitochondrial function, ultimately relieving cell-cycle arrest via the Cyclin D1/Rb axis. Solid arrows indicate connections directly demonstrated by experimental evidence in this study (ADCY5 upregulation by RNA-seq/qPCR; cAMP elevation by ELISA; CREB phosphorylation by Western blot; autophagy activation by LC3-II and chloroquine flux assays). Dashed arrows indicate inferred or previously reported connections that were not directly tested in this study (e.g., ATP→cAMP, cAMP→PKA, PKA→CREB, CREB→FXR-CREB/CRTC2). Dotted lines indicate downstream functional outcomes. Images created with BioRender.com, under permission. Data are presented as mean ± SD from at least three independent experiments. ns, not significant; *p < 0.05, **p < 0.01, ***p < 0.001 versus the respective control group.

### 3.7 Proposed mechanism of melissic acid-mediated alleviation of SHED senescence

Based on the findings above, we propose a mechanistic model in which the phytochemical melissic acid upregulates ADCY5, leading to increased cAMP production and activation of CREB phosphorylation at Ser133. Activated CREB promotes autophagy-related signaling, which is associated with improved lipid homeostasis, reduced peroxidative damage, and restoration of mitochondrial function. These changes, in turn, relieve senescence-associated cell-cycle arrest through activation of the Cyclin D1/Rb axis, ultimately contributing to the amelioration of senescent SHED phenotypes and recovery of cellular function. A schematic illustration of this proposed mechanism is presented in Figure 6G.

## 4 Discussion

Cellular senescence is increasingly recognized as a major driver of organismal aging and age-related tissue dysfunction. In the present study, we identified melissic acid, a natural long-chain saturated fatty acid, as a candidate senomorphic compound through phenotypic screening of 800 natural small molecules. Using both replicative and doxorubicin-induced senescence models in SHED, we demonstrated that melissic acid alleviated canonical senescence phenotypes, reduced DNA damage signaling, improved mitochondrial function, and promoted cell-cycle re-entry without causing overt cytotoxicity. Transcriptomic profiling further revealed that melissic acid prominently upregulated ADCY5 and coordinated transcriptional programs related to cAMP signaling, autophagy, and lipid metabolism. Mechanistically, melissic acid increased CREB phosphorylation, enhanced Beclin-1 expression, reduced p62/SQSTM1 accumulation, and suppressed lipid peroxidation, ultimately supporting reactivation of the Cyclin D1/Rb axis. Collectively, these findings suggest that the phytochemical melissic acid counteracts senescence in SHED through activation of an ADCY5/cAMP/CREB-dependent autophagic pathway.

A notable strength of this study is the use of phenotypic screening to identify a natural compound with senomorphic potential. Compared with target-based approaches, phenotypic screening provides an unbiased strategy for discovering compounds whose mechanisms may not be anticipated in advance. Phytochemicals and other natural products remain an important reservoir for senotherapeutic discovery because of their structural diversity and generally favorable biocompatibility (Yousefzadeh et al., 2018; Smiley et al., 1991). Several plant-derived compounds, including fisetin and quercetin, have been reported to exhibit anti-senescence activity; however, most known senolytics act primarily through apoptosis-related mechanisms by disrupting senescence-associated anti-apoptotic pathways (SCAPs) (Zhu et al., 2017). In contrast, our data suggest that melissic acid exerts its effects through metabolic remodeling and organelle quality control rather than direct induction of apoptosis, highlighting a potentially distinct mode of senescence modulation. A critical distinction in senotherapeutics is between senolytic agents, which selectively induce apoptosis in senescent cells (Kirkland et al., 2017), and senomorphic agents, which modulate senescence-associated phenotypes without eliminating cells (Xu et al., 2021). Our TUNEL data confirm that melissic acid does not induce apoptosis in senescent SHED (Supplementary Figure S1), consistent with the minimal cytotoxicity observed in CCK-8 assays (Figure 1F). These findings, together with the observed reduction in SA-β-Gal positivity and restoration of cell-cycle re-entry, collectively support the classification of melissic acid as a senomorphic rather than senolytic agent.

Transcriptomic profiling identified ADCY5 as one of the most strongly upregulated genes after melissic acid treatment, suggesting a previously unrecognized role for adenylate cyclase 5 in senescence modulation. ADCY5 catalyzes cAMP generation and thereby links extracellular cues to the cAMP/PKA/CREB signaling cascade. cAMP/CREB signaling is known to regulate transcriptional programs involved in metabolism, stress adaptation, and cellular homeostasis. Notably, CREB can promote transcription of autophagy-related genes and has been implicated in coordinating stress responses that preserve organelle quality (Seok et al., 2014; Ning et al., 2023; Zhu et al., 2015). In our study, increased CREB Ser133 phosphorylation, together with elevated Beclin-1 and reduced p62/SQSTM1, is consistent with activation of a CREB-dependent autophagic program. Direct measurement of intracellular cAMP by ELISA confirmed that melissic acid significantly elevates cAMP levels in senescent SHED (Supplementary Figure S2), providing biochemical evidence for ADCY5/cAMP pathway activation. While loss-of-function validation of ADCY5 or CREB was not performed, the combined transcriptomic and protein data support ADCY5/cAMP/CREB as a plausible upstream axis mediating melissic acid activity.

Autophagy is a critical quality-control pathway that maintains cellular homeostasis by removing damaged proteins, lipids, and organelles. Dysfunction of autophagy is closely associated with aging and cellular senescence, while restoration of autophagic activity has been shown to mitigate age-related decline (Seok et al., 2014; Levine and Kroemer, 2019). In senescent cells, defective autophagy can contribute to the accumulation of damaged mitochondria, oxidative stress, and persistent inflammatory signaling (Green and Levine, 2014). Consistent with this paradigm, we found that melissic acid reduced lipid peroxidation and restored mitochondrial membrane potential in senescent SHED. These findings suggest that melissic acid may alleviate senescence-associated oxidative stress by enhancing organelle quality control and membrane homeostasis, processes that are directly relevant to the topic of mitochondrial dysfunction and oxidative stress in age-related diseases. The decrease in p62/SQSTM1 further supports increased autophagic cargo turnover, although definitive confirmation of autophagic flux will require additional experiments using lysosomal inhibitors.

An observation that warrants discussion is the modest reduction of p53 expression in non-senescent SHED upon melissic acid treatment (Figure 3B). p53 is a critical tumour suppressor that maintains genomic integrity; its downregulation in normal cells could theoretically increase susceptibility to genomic instability. However, several factors should be considered when interpreting this finding. First, the reduction was partial and occurred in a concentration-dependent manner. Importantly, p21, a key downstream effector of p53, remained essentially unchanged in non-senescent cells at all tested concentrations (Figure 3B), suggesting that the functional p53-p21 axis was not significantly compromised. Second, no overt cytotoxicity or abnormal proliferation was observed in melissic acid-treated P6 cells (Figure 1F), indicating that the cells maintained normal growth control. Third, the reduction was most pronounced at the highest concentration (20 μM), whereas the major mechanistic findings were obtained at 10 μM, where the effect on normal cells was less pronounced. Nonetheless, we acknowledge that any modulation of p53 in normal stem cells warrants caution. Future studies will assess the long-term effects of melissic acid on genomic stability in non-senescent cells, including γ-H2AX foci kinetics, micronucleus formation, or comet assays, to comprehensively evaluate its safety profile.

Another important observation is that melissic acid relieved G0/G1 arrest and promoted cell-cycle re-entry in senescent SHED. Senescence is typically maintained by stable growth arrest mediated by the p16/Rb and p53/p21 pathways (López-Otín et al., 2023). In our study, melissic acid decreased p16, p21, and p53 expression, increased Cyclin D1 expression, and enhanced Rb phosphorylation at Ser780. These changes indicate partial restoration of proliferative signaling and reactivation of the Cyclin D1/CDK4/Rb axis. We interpret this finding cautiously, because cell-cycle re-entry in a mixed cell population may reflect selective recovery of non-senescent cells or escape from growth arrest by a subset of stressed cells rather than complete reversal of senescence in all cells. Nonetheless, the coordinated reduction of senescence markers and restoration of cell-cycle regulators strongly supports an anti-senescent effect of melissic acid (Montminy, 1997; Nakahira et al., 2011; Hamsanathan and Gurkar, 2022).

SHED represent a relevant model for studying stem cell exhaustion, a key cellular process in aging, due to their robust proliferative capacity and regenerative potential. Controlling SHED senescence is therefore critical for improving stem cell-based therapies, as senescence impairs their self-renewal, differentiation, and therapeutic efficacy. Our findings suggest that melissic acid-mediated senomorphic modulation could enhance SHED fitness prior to therapeutic application. Thus, identifying melissic acid as a senomorphic candidate in SHED has implications for both aging biology and regenerative medicine. A natural compound that selectively improves the quality of senescent SHED while sparing non-senescent cells may be valuable for stem cell therapy and for optimizing expanded stem cell populations.

Nevertheless, this study has several limitations. First, while our pharmacological inhibition experiments using SQ22536 confirm that ADCY5 is required for melissic acid’s anti-senescence effects, genetic knockdown experiments (e.g., siRNA for ADCY5 or CREB) were not performed. Future studies employing genetic perturbation would further strengthen the causal evidence for the ADCY5/cAMP/CREB axis. Second, while our LC3B and chloroquine flux assays confirm that melissic acid enhances autophagic flux (Supplementary Figure S5), we acknowledge that p62 dynamics in the context of chloroquine co-treatment were not examined. Future studies will address this to further delineate the interplay between autophagic flux and p62 turnover. Third, we acknowledge that the RNA-seq experiment was performed with only two biological replicates per condition (n = 2), which limits statistical power and precludes robust differential expression analysis based solely on RNA-seq data. However, all candidate genes identified from this screening were validated by qPCR with three independent biological replicates (n = 3), and the mechanistic conclusions of this study are primarily based on these validated data. Future studies with larger replicate numbers would further strengthen the transcriptomic analysis. We acknowledge that cell cycle analysis was performed only in the replicative senescence (P20) model and was not included for the ADR-induced senescence model in the current study. While cyclin D1 upregulation and Rb phosphorylation were consistently observed in ADR-treated cells (Figures 5C,D), providing indirect support, direct flow cytometric confirmation of cell-cycle re-entry in the ADR model will be addressed in future studies to establish full consistency across both senescence models. To further distinguish between true phenotypic reversal and selective outgrowth of non-senescent subpopulations, future studies will employ EdU incorporation combined with SA-β-Gal staining. This approach will allow direct visualization of whether proliferating cells (EdU-positive) arise from the senescent population (SA-β-Gal-positive), providing single-cell resolution evidence for the mechanism of action of melissic acid.

It is important to acknowledge that melissic acid is a C30 straight-chain saturated fatty acid with very low aqueous solubility. In the present *in vitro* study, we used DMSO as a co-solvent and relied on the culture medium for compound dispersion, which is a standard approach for delivering lipophilic compounds in cell-based assays. The stable, reproducible, and concentration-dependent biological effects observed across multiple independent experiments provide indirect evidence that melissic acid was effectively available to the cells under our experimental conditions. However, achieving 5–20 μM tissue concentrations *in vivo* would likely require specialized formulation strategies, such as nanoemulsions, lipid-based carriers, or cyclodextrin inclusion complexes, to enhance oral bioavailability and systemic distribution. While our findings provide proof-of-concept evidence for the senomorphic activity of melissic acid, further pharmacokinetic and formulation studies are necessary to assess its translational potential. We therefore caution against over-interpreting the *in vivo* relevance of our current results, and we have revised the Conclusions accordingly to reflect this limitation.

In conclusion, this study identifies melissic acid as a natural senomorphic candidate that alleviates senescence-associated phenotypes in SHED through a mechanism involving ADCY5/cAMP/CREB activation, autophagic-associated metabolic remodeling, and restoration of cell-cycle progression. These findings broaden the mechanistic landscape of phytochemical-based senotherapeutics and suggest that targeting the ADCY5/cAMP/CREB-autophagy axis may represent a promising strategy for senotherapy and stem cell rejuvenation, thereby contributing to the growing field of phytochemical anti-aging interventions.

## Data Availability

The data presented in the study are deposited in the NCBI Sequence Read Archive (SRA) repository, accession number PRJNA1526383, SRR40595915.
